# Quantitative determination of environmental factors governing the snow melting: a geodetector case study in the central Tienshan Mountains

**DOI:** 10.1038/s41598-022-15722-5

**Published:** 2022-07-07

**Authors:** Haixing Li, Jinrong Liu, Xuelei Lei, Yumeng Ju, Xiangxu Bu, Hongxing Li

**Affiliations:** 1 College of Geomatics Science and Technology, Nanjing Tech University, Nanjing, 211816 China; 2 National Cryosphere Desert Data Center, Lanzhou, 730000 China; 3 Geospatial Information Research Center, Nanjing Tech University, Nanjing, 211816 China; 4 Institute of Remote Sensing and Image Processing, Nanjing Tech University, Nanjing, 211816 China

**Keywords:** Environmental impact, Cryospheric science

## Abstract

Because of the distinctive vertical climate and topography gradients in the alpine region, the snow cover of the Tienshan Mountains possesses complex spatiotemporal heterogeneity, particularly during the melting process. Quantifying the environmental factors is therefore crucial to understanding the melting process and for predicting and managing snowmelt runoff. Herein, the snow cover area, grain size, and contamination extent were determined to characterize the detailed melting status based on surface reflectance data of MOD09A1 in the central Tienshan Mountains from 2013 to 2017. The environmental factors collected include relief (elevation, slope, and aspect); meteorology (surface air temperature, land surface temperature, solar radiation, and wind speed); and land surface vegetation. Analysis of the geodetector results indicated the following. (1) Patterns of changes in the overall dominant environmental variables were consistent for the pre-, mid-, and post-melting periods defined according to the decline of snow cover area over five years. (2) The overall major environmental factors were wind speed and radiation (pre-period), land surface temperature and elevation (mid-period), and elevation and land surface types (post-period), respectively. (3) Regional distinctions were detected of the dominant environmental factors. In the pre-melting period, the effects of solar radiation and wind speed were noticeable in the north and south regions, respectively. The effects of elevation, land surface temperature, and land cover types became more prominent in all regions during the mid- and post-melting periods. (4) Interaction between the major environmental factors was significantly enhanced on both the overall and regional scales, thus affecting the snow-melting process. Finally, the energy distribution mismatch resulted in the snowmelt. Multiple environmental factors substantially affect heat redistribution at different spatiotemporal scales, resulting in the snowmelt as a complex manifestation of the factors and their interactions. The findings highlight regional differences in various environmental factors of the melting process and offer a theoretical foundation for the melting process at various scales over multiple years.

## Introduction

As one of the most active natural components and a crucial parameter of the cryosphere, the snow cover exerts a considerable effect on moisture and energy exchanges between the land surface and the atmosphere owing to its particular physical features (e.g., high emissivity, high albedo, large latent heat of melting, and low thermal conductivity)^1,2^. Because of large-scale airflow from north to south and from west to east, along with small-scale precipitation regimes, the Tienshan Mountain Range located in the central Eurasian continent has abundant snow accumulation^3^. With the large snow cover, this area serves as the “water tower” of Central Asia, which is the main water source for the nearby oasis and desert ecosystem^4,5^. However, the annual temperature variation in northwestern China constantly affects the snowfall regimes and runoff patterns supplied in spring and summer to this area^6^. The significant differences in altitudinal structure and land-use types, as well as the effects of radiation, precipitation gradients, forest belts, and soil moisture content complicate the spatiotemporal distribution of snow cover^7,8^. Thus, this area displays complex distribution patterns of snowpack types and diverse influence factors across climate zones, altitude belts, temperature and vegetation ecosystems, and eco-geographical zones^9,10^.

Numerous studies have demonstrated significant spatiotemporal difference in the environmental factors that affect snow accumulation and the melting process. These factors are generally distinguished by macro-scale factors such as climate change^2,11^ and micro-scale factors such as relief (elevation, slope, and aspect), meteorology (temperature, radiation, and precipitation), and vegetation. For example, it has been confirmed that alterations in the Central Asian water balance are due to changes in macro-climate and human interaction, resulting in differences in the interannual phenological characteristics of snow cover on an intercontinental scale^12^. Macro-climate effects can also manifest in the form of microclimates that transform the local phenological characteristics of snow cover within subregions^13–15^.

While catchment-scale studies have determined topography to be the main environmental factor affecting snow accumulation and melting^16–18^, such altitude dependence is expressed as significant positive feedback between various snow phenology characteristics and altitude, resulting in distinct values of snowfall, snow depth, and snow cover duration in different altitude belts^1,18^.

The effect of local meteorological factors (such as precipitation, air temperature, and radiation) on the spatiotemporal snow cover distribution differs by altitude zone^19–21^. For example, decreased snowfall and increased rainfall and temperature have been identified as the primary reason for decreases in snow cover duration and snow water equivalent in the Tibetan Plateau^18^. Similar conclusions were reached for the Heihe River Basin^22^. In-field measurement results obtained there confirmed that radiation provided nearly all the energy for snow melting in high-altitude areas^23,24^.

The influence of land surface vegetation on snow accumulation and ablation is also noticeable. Different vegetation types characterized by varying evapotranspiration and water storage behaviors affect the density, water content, depth, and other snow parameters, determining the snow ablation characteristics^25^. This relationship between the spatiotemporal pattern of alpine vegetation phenology and snow cover dynamics is bidirectional^21,25^.

Altogether, the aforementioned conclusions indicate that under complex compound interactions between macro- and micro-scale factors, both the snow cover parameters and the environmental factors exhibit heterogeneity at different scales^4,19^. Therefore, accurately determining these environmental factors is crucial for understanding the regional hydrological processes and mechanisms of influence. Numerous studies that determine the dynamics of independent variables have been conducted by exploring spatiotemporal changes in snow parameters under different influencing factors, thereby indirectly highlighting key factors of snow accumulation or ablation at different temporal and spatial scales^26,27^. The classical statistical methods and spatial statistical models, such as principle component, multivariate analyses, geospatial probabilistic occurrence ratio and geographically weighted regression models, have been widely used to assess snow variations^24,28–31^. However, the number of variables considered in these models are limited, and the models cannot quantify the contribution of different environmental factors and they do not consider the multivariate collinearity problem.

The geodetector method^32^ hypothesizes that a dependent variable will exhibit a similar spatial or temporal distribution to that of the correlated variables and can be used to diagnose interactions between the latter. Because of its tremendous capacity to use information regarding different types of influencing factors, this geospatial technique is extremely useful for identifying environmental factors of snow melting. The study area chosen for this work was the central Tienshan Mountain Range. A quantitative analysis of environmental factors governing the snow-melting was performed using a geodetector method by combining snow status information and multi-source variables from 2013 to 2017. To the best of our knowledge, the geodetector model has not yet been used to interpret snow cover variation dynamics or the related variables, which constitutes a novel contribution of the present study.

## Study area and data source methodology

### Study area

**Figure 1:**
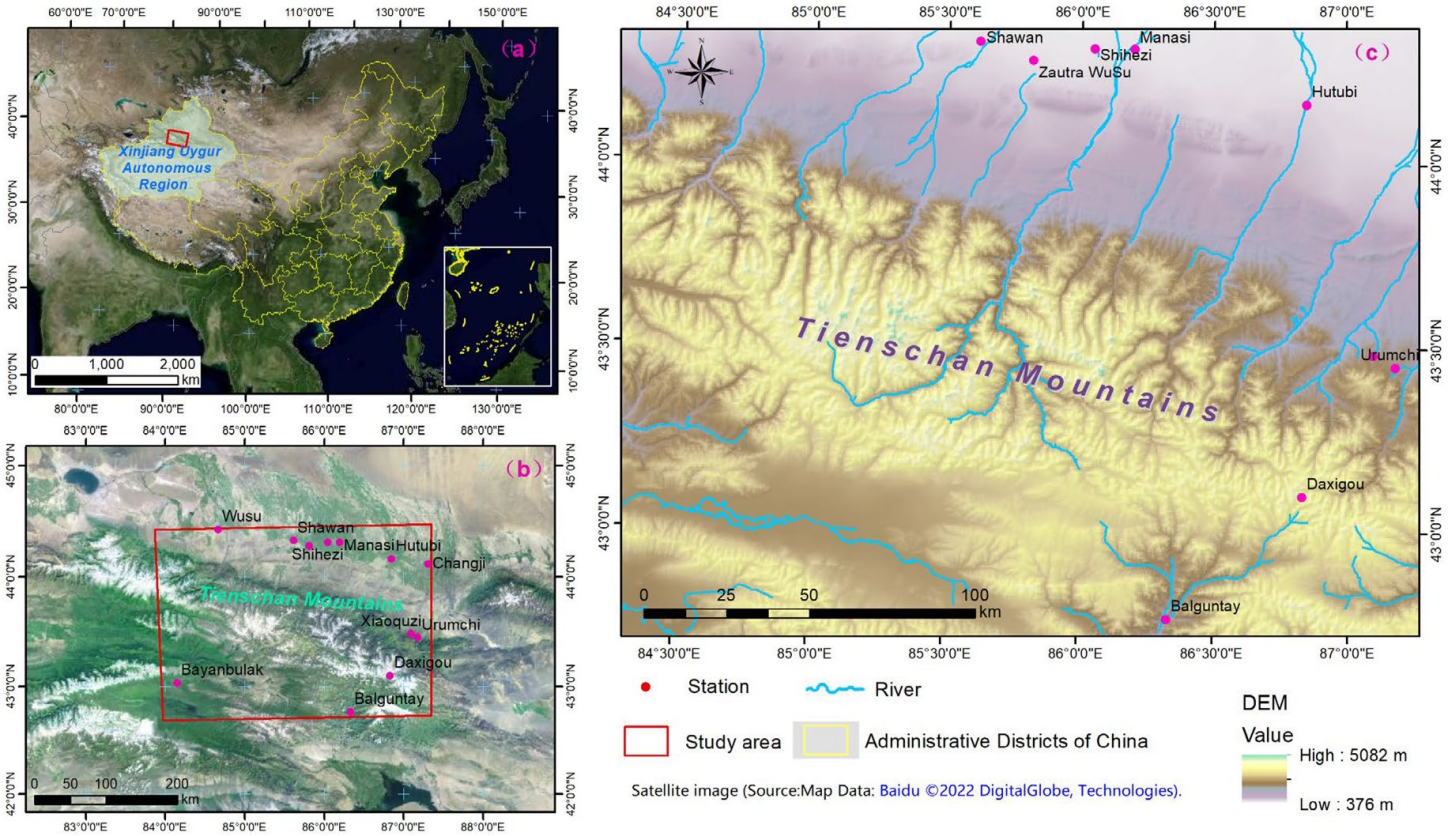
Location of the study area. (**a**,**b**) show the location of study area within China and Tianshen mountains with the satellite image obtained from Baidu ©2022 DigitalGlobe, Technologies (https://map.baidu.com/search/%E5%85%A8%E5%9B%BD/@12959219.599999992,4825334.641182318,5.33z/maptype%3DB_EARTH_MAP?querytype=s&da_src=shareurl&wd=%E4%B8%AD%E5%9B%BD&c=224&src=0&pn=0&sug=0&l=18&b=(13432344.2131852,3651617.4721446065;13433629.447638426,3652204.281511593)&from=webmap&biz_forward=%7B%22scaler%22:1,%22styles%22:%22sl%22%7D&device_ratio=1). The red dots are the meteorological stations. (**c**) is the elevation map that obtained from Resource and Environment Science and Data Center (https://www.resdc.cn/Default.aspx). Map created in ArcMap 10.6 of the Environmental System Resource Institute, Inc. (https://www.esri.com/software/arcgis/arcgis-for-desktop). Boundaries made with free vector data provided by National Catalogue Service for Geographic Information (https://www.webmap.cn/commres.do?method=dataDownload).

The study area is located in the central area of the Tianshan Mountains, in the heart of the Eurasian continent, within an area bounded by 83.57°E, 87.50°E, 42.41°N, and 44.28°N (Fig. 1). It stretches from the west–southwest to east–northeast and occupies approximately 538,000 km^2^, covering portions of the Xinjiang Uyghur Autonomous Region in China. The altitude ranges from 376 to 5082 m a.s.l., with some elevations exceeding 5000 m a.s.l. The land cover types include grassland (58.7%), barren and sparsely vegetated regions (32.8%), cropland (3.7%), forest (0.4%), urban (0.25%), and scrublands (0.03%).

This area is dominated by a typical temperate continental arid and semi-arid climate, which is characterized by temperature extremes in summer and winter. Within this area, the absolute maximum and minimum temperatures are 40 °C and 38 °C, respectively, with an annual average temperature of 6.2–7.8 °C. The annual accumulated temperature over 10 °C is 2400–3500 °C. At 3800 m above sea level, the air temperature gradually decreases with the elevation and stays below 0 °C year-round and above the snow line^33^. The surrounding deserts and dry areas reflect the characteristic aridity of the region. Majority precipitation falls on the windward western and northwestern slopes that are exposed to cold northerly and northwesterly air inflows, as well as moist westerly influxes from the North Atlantic. Precipitation amounts vary from 150–220 mm up to 600 mm^34^.

### Data collection

Herein, two datasets were collected and used as independent and dependent variables: the snow cover index dataset representing the status of snowpack and the environmental factor dataset, including multi-source potential influencing factors (Table 1). The change in the snow- cover area is insignificant, especially early in the melting period and at the end of ablation. Thus, the de-cloud daily reflectance data were selected in our study to extract the slight variation of snow cover change. The snow cover index dataset was primarily obtained from the surface reflectance data from MOD09GA and the snow cover area (SCA) product from the MOD10A1 dataset. The 8-day synthetic SCA product from MOD10A2 was used to provide a long time-series and high-precision spatial snow distribution information. To reduce the effect of cloud on the inverse performance, the 8-day synthetic cloud-removing surface reflectance product from MOD09A1 was used to extract the snow state information.

**Table 1:** Description of potential snow melting predictor variables used in this study.

Variables types	Factor types	Variable name	Data source	Spatial resolution	Time resolution	Unit	Download links
Dependent variable factors	Snow status parameters	Snow grain index	MOD09GA	500 m	8 days	–	https://ladsweb.modaps.eosdis.nasa.gov/
		Snow contamination index					
	Snow cover product	Snow-covered area	MOD10A1	500 m	Daily	–	http://www.crensed.ac.cn/portal/
Independent variables factors	Terrain Relief	Elevation	ASTER G-DEM	90 m	–	M	http://gdem.ersdac.jspacesystems.or.jp/
		Slope				°	
		Aspect				°	
	Organism	Land cover types	MCD12Q1	500 m	Annual	–	https://ladsweb.modaps.eosdis.nasa.gov/
	Meteorology	Land Surface Temperature	AWLSTD	1 km	Daily	K	https://data.tpdc.ac.cn/zh-hans/
		Near-surface temperature (2 m)	CMFD	0.01°	Daily	K	https://data.tpdc.ac.cn/en/data
		Downward shortwave Radiation		0.01°	Daily	W/m^2^	
		Near ground full wind speed (2 m)		0.01°	Daily	m/s	

To reduce the effect of clouds on surface temperature, we used the all-weather land surface temperature dataset (AWLSTD) produced by merging satellite thermal infrared and passive microwave observations^35^. This merging method was applied to MODIS and AMSR-E/AMSR2 data to produce a multi-year record of 1-km all-weather land surface temperature (LST) values over western China.

The environmental factors dataset comprised the following: (1) terrain factors, such as elevation, slope, and aspect calculated and resampled by a digital elevation model (DEM) data with 90-m resolution. These data can be downloaded from the Shuttle Radar Topography Mission website (http://srtm.csi.cgiar.org/); (2) meteorological factors, including air temperature, solar radiation, and precipitation data, which were extracted from the China meteorological forcing dataset (CMFD)^36^; (3) LST data obtained from the AWLSTD over western China.

Previous work has shown the CMFD, which includes precipitation data, assimilation of precipitation products from remote sensing, the Tropical Rainfall Measuring Mission (TRMM) and conventional meterological observation data, to be suitable for ground meterological elements in regional in China with high spatiotemporal resolution^37^. Compared with other datasets, it has the advantage of higher accuracy and time sequences for mountainous areas lacking sufficient weather stations.

### Data preprocessing

All datasets were projected onto an Albers conical equal area WGS1984 coordinate system. Because the spatial resolution of the terrain and climate factors do not perfectly match those of the snow status and SCA data, all terrain and climate parameters need to be processed to match the snow cover data.

**Figure 2:**
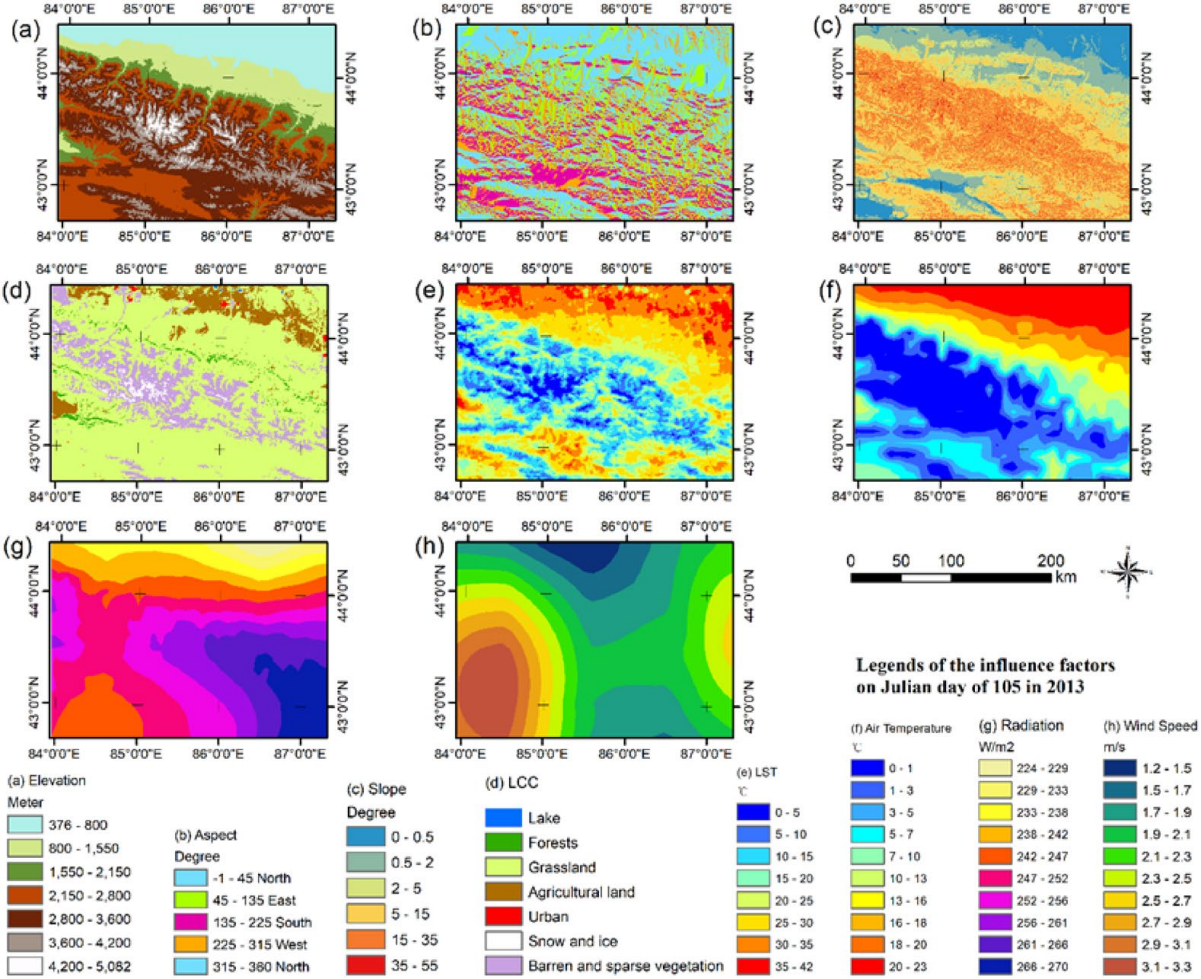
Spatial distributions of all environmental factors in the study area. (**a**–**c**) are the factors of elevation, aspect and slope calculated by the DEM dataset (https://www.resdc.cn/Default.aspx). (**d**) is the factor of Land cover classification (https://ladsweb.modaps.eosdis.nasa.gov/). (**e**) is the factor of land surface temperature of the dataset of AWLSTD (https://data.tpdc.ac.cn/zh-hans/). (**f**–**h**) are the factors of air temperature, the shortwave radiation, and the wind speed from the dataset of CMFD. (https://data.tpdc.ac.cn/en/data). Map created in ArcMap 10.6 of the Environmental System Resource Institute, Inc. (https://www.esri.com/software/arcgis/arcgis-for-desktop).

The reflectance data and SCA products selected in this study are both 8-day composite data, and the effect of cloud content is negligible. Because the geodetector method only handles discrete variables, we converted the eight continuous variables into discrete versions. In combination with expert knowledge, we classified the elevation of the northern slope of the TRM according to the vertical vegetation zones. We classified the slope and aspect according to the existing standards^34^. Because of the large differences in surface temperature, near-surface air temperature, downward radiation, and wind speed among different dates, the equal interval method was used to fine-grade all of the factors. A classification scheme for plant functional types was used to select the land cover data from the annual scientific datasets MCD12Q1 from 2013 to 2017. The land cover product was reclassified into broad types: forests, grasslands, agricultural land, urban area, snow and ice, and barren and sparse vegetation. The grading standards of various factors are shown in Fig. 2.

## Methodology

The methodology used in this study comprises three steps. First, the SCA was extracted from three snow cover indexes along with the melting status of each grain size and the contamination extent. Second, the melting season was divided into three recession periods in each year according to the SCA variations. The study area was also divided into several partitions based on merging areas in common watershed basins. Finally, the geodetector model was implemented to quantitatively determine the impact factors of snow melting on the overall and regional scales.

### Retrieval of snow melting parameters

The NDSI has been widely used to differentiate between snow and non-snow pixels using green and shortwave–infrared bands, respectively^38^. It may deal with topographic effects^39^, such as delineating and mapping the snow in mountain shadows^40^. The albedo was found to decrease with the effective increase in grain size because of the clustering of snow crystals. The snow grain index is a normalized difference index of reflectance with green and near-infrared red bands that helps to show grain size variation^39^. A high SGI value indicates a large particle size. Similarly, the maximum effect of snow contamination is observed in the visible region (0.3 and 0.7 μm) and decreases with increasing wavelength (decreases up to 1.3 μm and is negligible beyond 1.3 μm). The value of the SCI remains negative for all types of contamination. Therefore, using cloud-free reflectance for each hydrological year from January 2013 to June 2017, we retrieved optical snow cover indexes for each pixel, including the normalized difference snow index (NDSI)^38^, the snow grain index (SGI), and the snow contamination index (SCI)^39–42^. NDSI, SGI, and SCI all range from − 1 to + 1. The snow cover metrics were calculated by overlaying these indexes on the SCA extracted from the MOD10A1 data, which serves as the dependent variable in our study.

1 $${\text{NDSI}} = \frac{{\left( {Reflectance\left( {590\;{\text{nm}}} \right)} \right) - \left( {reflectance\left( {1040 - 1050\;{\text{nm}}} \right)} \right)}}{{\left( {Reflectance\left( {590\;{\text{nm}}} \right)} \right) + \left( {reflectance\left( {1040 - 1050\;{\text{nm}}} \right)} \right)}}.$$

2 $${\text{SGI}} = \frac{{\left( {Reflectance\left( {590\;{\text{nm}}} \right)} \right) - \left( {reflectance\left( {1040 - 1050\;{\text{nm}}} \right)} \right)}}{{\left( {Reflectance\left( {590\;{\text{nm}}} \right)} \right) + \left( {reflectance\left( {1040 - 1050\;{\text{nm}}} \right)} \right)}}.$$

3 $${\text{SCI}} = \frac{{\left( {Reflectance\left( {470\;{\text{nm}}} \right)} \right) - \left( {reflectance\left( {590\;{\text{nm}}} \right)} \right)}}{{\left( {Reflectance\left( {470\;{\text{nm}}} \right)} \right) + \left( {reflectance\left( {590\;{\text{nm}}} \right)} \right)}}.$$

### Spatiotemporal partitioning of the snow melting process

In our study, to accurately determine the spatiotemporal heterogeneity of influencing factors, the snow melt season was divided into recession periods and the study area was partitioned into several subregions.

#### Division of snowmelt recession periods

**Figure 3:**
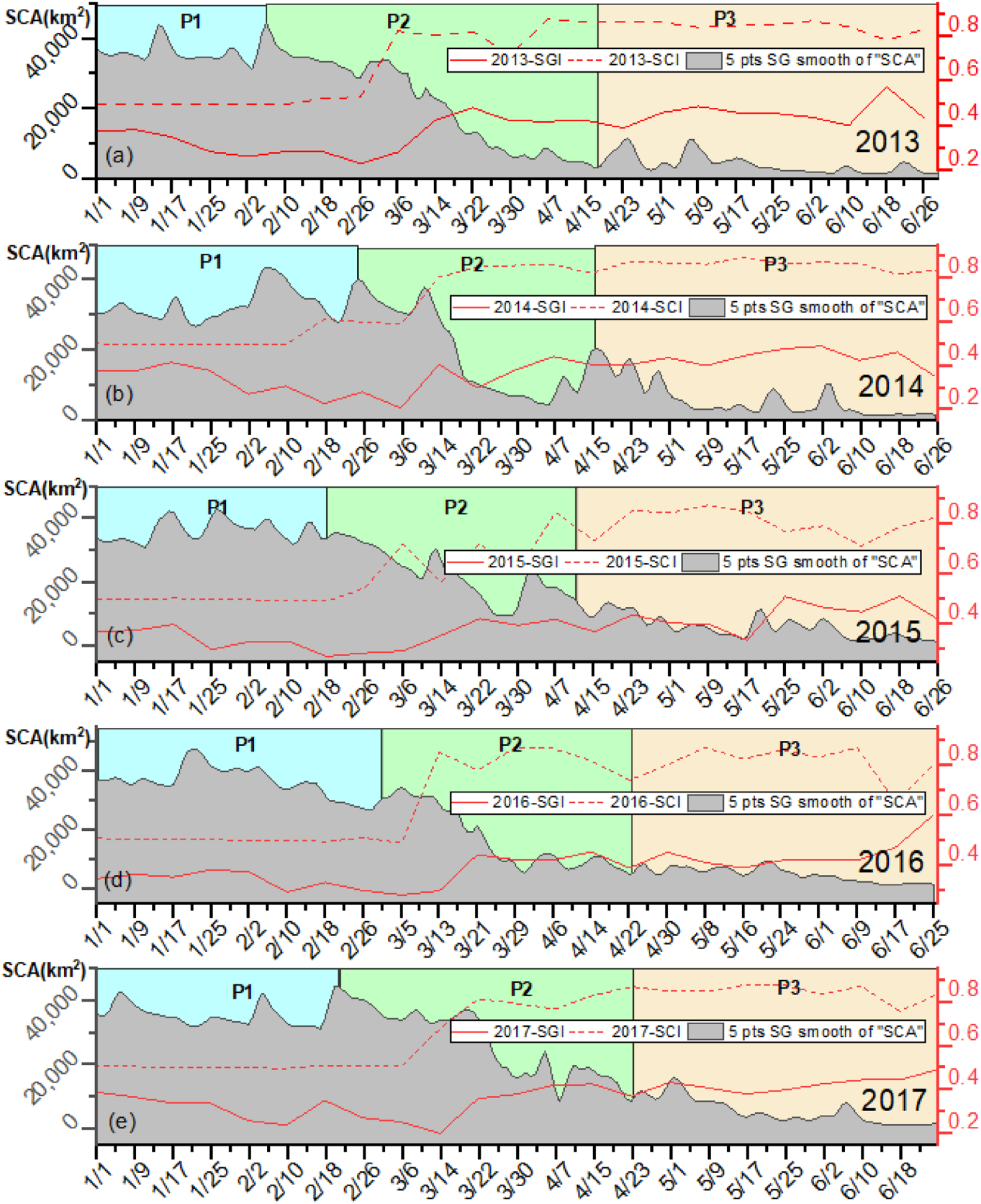
Variations in the SCA, SGI, and SCI during snowmelt seasons from 2013 to 2017. Map created in OriginPro, Version 2021. OriginLab Corporation, Northampton, MA, USA. (https://www.originlab.com/).

Owing to the limited number of available meteorological stations in the study area, the declining SCA curves during the melting season were used to subdivide the snow cover ablation period. The Savitzky–Golay filtering method was used to smooth the SCA decline curve to reflect the overall snow recession trend. The general process of snow recession can be approximated as a cosine curve. There is a process of slow decay, rapid decline, and a slow decline again. Therefore, we refer to the snow decline curve above and divide the process into three stages^43^. The slope of the smoothed SCA curve was used to roughly divide the period of snow cover decline on a year-by-year basis to achieve a detailed analysis of the snowmelt process. Figure 3 depicts the smoothed SCA curve after Savitzky–Golay filtering and the three periods defined as pre-melting (P1), mid-melting (P2), and post-melting (P3). The changes in the SGI and SCI curves during snowmelt periods are displayed as well.

#### Spatial partitioning of the snow melting process

Watershed topographic features were extracted using a DEM to delimit the watersheds in the research area. The ArcGIS hydrological toolbox was used to pre-fill the terrain, extract flow directions, calculate the flow accumulation, extract the river networks and water systems, divide the grades, and define the range of sub-basins. As shown in Fig. 6, all the sub-basins were merged into the following six subregions: (1) the lower reaches of the northern slope of the MTR, (2) the western side of the Manas River Basin, (3) the Manas River Basin in the MTR, (4) the eastern Manas River Basin, (5) the Bayanbulak area, and (6) the Kaidu River Basin.

### Discretization of environmental factors data

The continuous data for various environmental factors were discretized using selected classification algorithms to ensure good results from the geodetector model. Expert knowledge was used to discretize the factor elevation, while the equal-interval discrete method was used to classify other factors. As shown in Fig. 2, the eight continuous variables were discretized into 4–12 intervals through these discretization methods.

### Geographical detector models

This study implemented the geodetector method to attribute variations in snow parameters to related environmental factors at seasonal and annual scales. The geodetector method is a quantitative technique that determines whether the spatial distribution of a geostatistical variable is similar to that of an independent variable. This model is based on spatially stratified heterogeneity, which exists in this region if the variances in the subregions are smaller than the variance in the overall region. Assuming that the spatial distributions of the independent variable (snow cover status) and the dependent variable (impact factor) are consistent, a statistical correlation exists between them, revealing a causal relationship of snow melting in alpine regions^32^.

Within the geodetector model, the *Q* value was used to quantitatively determine the heterogeneity and autocorrelation of the dependent variable. The relationship between the dependent variable and its influencing factors was also determined. The factor detection model and the interaction detection model are two geodetector functions used in this paper. The formula of the geodetector model is expressed as follows:

4 $$Q_{Xi} = 1 - \frac{1}{{N\sigma^{2} }}\mathop \sum \limits_{j = 1}^{L} N_{{X_{ij} }} \sigma_{{X_{ij} }}^{2} ,$$

where *i* is the number of determinants; *X*_1_, *X*_2_, …, *X*_22_ are the influencing factors selected for this study; *j* = 1, …; *L* refers to the strata of snow cover status or factor *Xi*; $N_{{X_{ij} }}$ and *N* refer to the number of units in layer *j* and the entire study area, respectively; and $\sigma_{{X_{ij} }}^{2}$ and $\sigma^{2}$ indicate the variances of the total snow cover status in layer *j* and the entire region, respectively. $Q_{Xi}$ values range from 0 to 1, where a large value indicates significant spatial stratification heterogeneity. The contributions of different factors to snow cover melting were also explored.

### Interactive effect of impact factors

The interactive effect of two variables (*X*_1_ and *X*_2_) on changes in the snow melting process can be quantified by the *q* statistic. The algorithm primarily creates a new stratum by superimposing *X*_1_ and *X*_2_, denoted as *X*_1_ ∩ *X*_2_. It then compares the *Q* values of the three factors to determine the type of interaction between the factors. By comparing the values of *Q* (*X*_1_ ∩ *X*_2_) with the values of *Q* (*X*_1_) and *Q* (*X*_2_), the index can be used to assess the interactive effects of the two factors (*X*_1_ and *X*_2_). Furthermore, the interactive relationship can be interpreted in terms of five categories, listed in Table 2, by comparing the interactive *Q* value of the two factors with the *Q* value for each of the two factors.

**Table 2:** Categories of interactive relationships between two factors.

Interaction type	Description
Weaken, univariate	*Min* (*Q*(*X*_*1*_), *Q* (*X*_*2*_)) < *Q* (*X*_*1*_ ∩ *X*_*2*_) < *Max* (*Q*(*X*_*1*_), *Q*(*X*_*2*_))
Weakened, nonlinear	*Q* (*X*_*1*_ ∩ *X*_*2*_) < *Min* (*Q*(*X*_*1*_), *Q* (*X*_*2*_))
Enhanced, bivariate	*Q* (*X*_*1*_ ∩ *X*_*2*_) > *Max* (*Q* (*X*_*1*_), *Q* (*X*_*2*_))
Enhanced, nonlinear	*Q* (X1 ∩ X2) > *Q* (X1) + *Q* (X2)
Independent	*Q* (X1 ∩ X2) = *Q* (X1) + *Q* (X2)

## Results

### Spatiotemporal variations in snow status during the melting process

**Figure 4:**
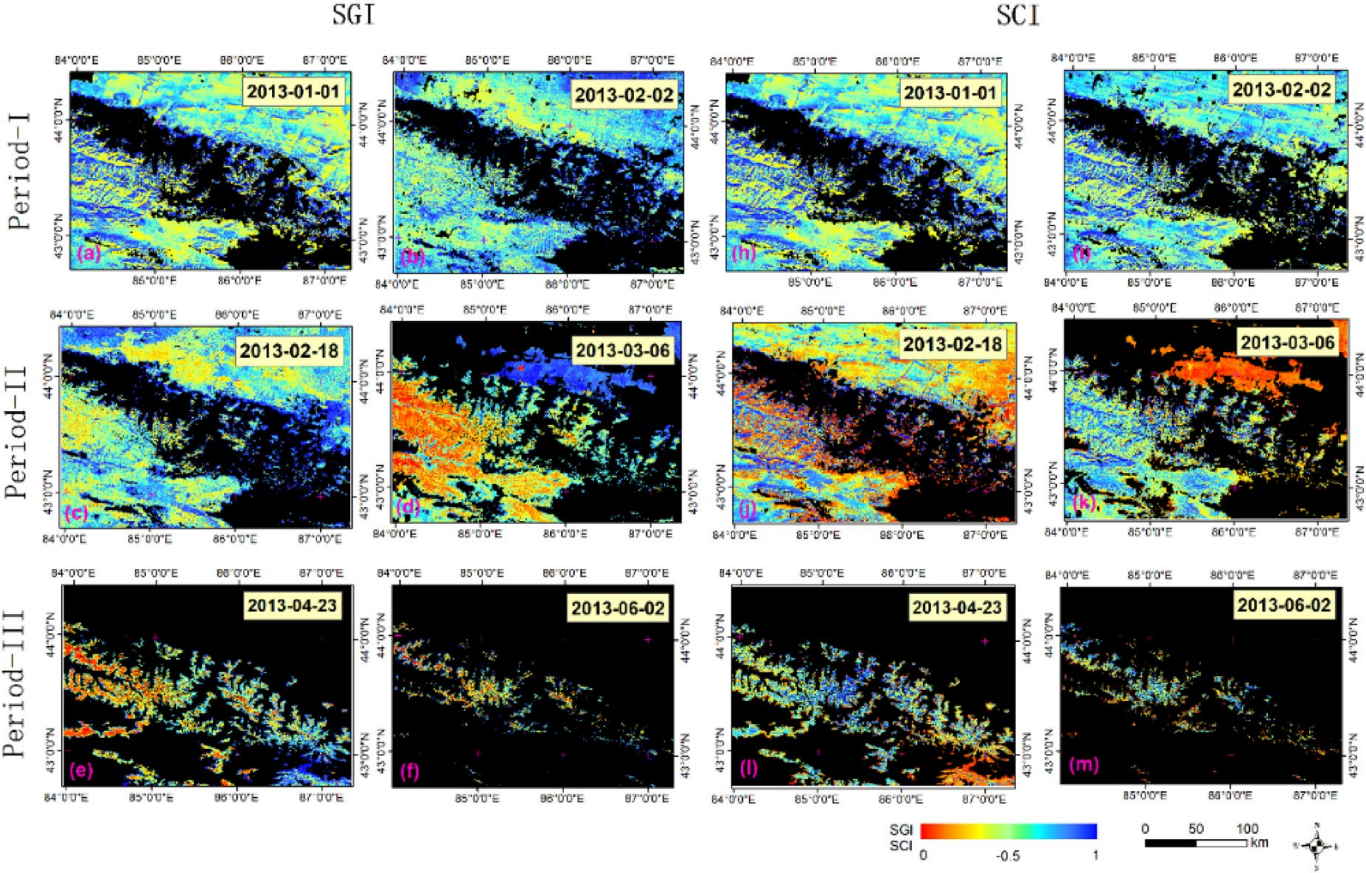
Spatiotemporal distributions of the SGI and SCI during snowmelt periods in 2013. (**a**–**f** and** h**–**m**) are the SGI and SCI that calculated from the reflectance data of MOD09GA (https://ladsweb.modaps.eosdis.nasa.gov/) and snow cover area data of MOD10A1 (http://www.crensed.ac.cn/portal/). Map created in ArcMap 10.6 of the Environmental System Resource Institute, Inc. (https://www.esri.com/software/arcgis/arcgis-for-desktop).

As shown in Fig. 3, the overall SCAs displayed a step-like decreasing trend from 2013 to 2017. The five-year average seasonal change in SCA was approximately 3500 km^2^, constituting over 90% of the total area, of which approximately 70% significantly decreased during the rapid ablation period. In contrast, both SGI and SCI displayed a growth trend in three stages. The increase in the SGI and SCI was inversely related to the step-like decrease in the SCA. The difference in annual changes between the SGI and SCI from 2013 to 2017 was insignificant over the five years. Figure 4 depicts the spatial distribution of the SCA, SGI, and SCI during snowmelt in 2013.

In the P1 stage, the entire research area was essentially covered by snow with a small grain size and contamination extent. In the P2 stage, the snow cover on northern piedmont slopes and that in low-altitude southern areas began to melt widely, with the snow grain size and contamination degree significantly increasing. The snow below the permanent snow line at the end of melting season almost disappeared, which is consistent with previoud studies of the snowline within Tienshan mountain^44–46^. The remaining snow exhibited a high degree of melting. Generally, the snow grain size and contamination degree are not consistent across time and space.

### Impact of influencing factors on the snow melting process

#### Overall dominant factors of snow melting

The factor detector in the geodetector model was used to analyze the major variation factors in the SGI and SCI. The *Q* value in the measurement results explains the extent to which each impact factor has caused spatial divergence in the two dependent variables (SGI and SCI). Table 3 shows the average *Q* values of the SGI and SCI for each influencing factor in each melt season from 2013 to 2017.

**Table 3:**
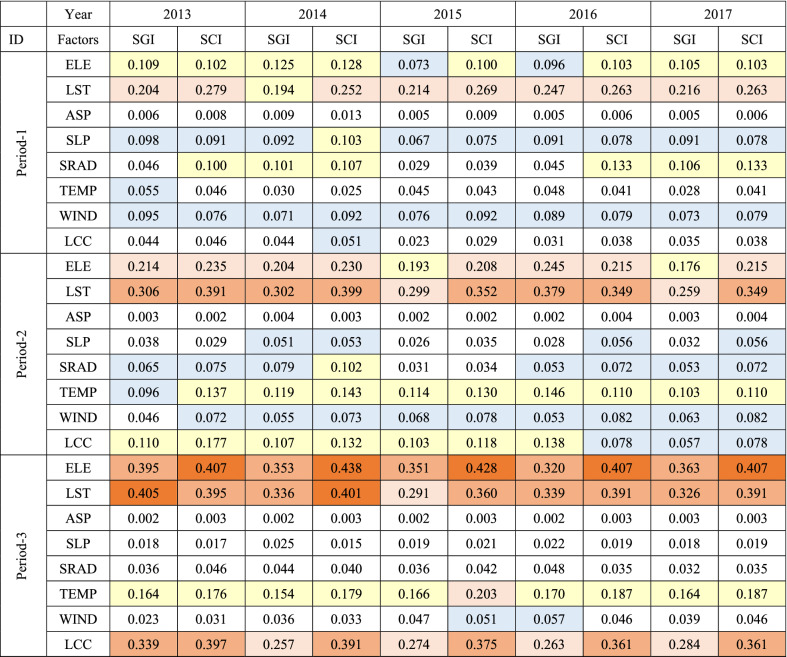
Average Q values of the SGI and SCI in periods-1, 2 and 3 from 2013 to 2017.

The warm and cool colors are used to distinguish the *Q* and *P* values. The warmer the color is, the more important it is, and vice versa.

Over the five years, the key influencing factors in different periods were relatively consistent. Accordingly, we speculated that the impact factors for the SGI and SCI in each period of the study area would exhibit similar patterns of temporal change over the five year period. The two most important influencing factors during the three periods were found to be LST and elevation (ELE), followed by wind speed (WSP), air temperature (TEMP), solar radiation (SRAD), and slope (SLP). We discovered that the order of the *Q* value for each factor appeared to be different in snowmelt periods when we compared the interannual variation characteristics of the *Q* value across three periods. Regardless, the order of the influencing factors remained consistent across years. The increase in the SGI and SCI was inversely related to the decrease in the SCA in a step-like fashion. The difference in annual changes between the SGI and SCI from 2013 to 2017 was insignificant over the five years. The spatial distribution of the SCA, SGI, and SCI on specific dates during snowmelt in 2013 is depicted in Fig. 4.

LST was the most dominant influencing factor of snow ablation during P1. The degree of influence of WSP, ELE, SRAD, and SLP was only 30% of that of LST and relatively similar. Note that the effect of TEMP was not obvious during this period. This is because during the early stage of melting, when the maximum daily temperature is lower than 0 °C, the SRAD and fluctuations in surface temperature it causes initiate a change in the snow cover state, while wind and terrain affect snow-cover reconstruction.

In P2, the effects of ELE as well as LST significantly increased. The effect of TEMP also increased significantly to reflect that of ELE. The effect of WSP and SRAD slightly decreased, whereas the effect of LCC remarkably increased. This may be because as the daily maximum temperature starts to rise above 0 °C, the effect of TEMP on snowmelt cannot be ignored. Moreover, the change in LCC accelerated the snowmelting process to a large extent. In comparison, WSP, SLP, ASP, and other factors did not obviously influence the snow state during this period.

In P3, ELE, LST, LCC, and TEMP were the leading factors of snow ablation with a significant degree of effect (*Q* >  ~ 0.4). The effects of WSP and ASP were weak enough to be ignored (*Q* <  ~ 0.01). This is because the snow cover gradually melts away with a gradual increase in temperature at the end of ablation. The remaining snow cover presents a typical vertical distribution feature, explaining why LCC displays a high spatial consistency with the snow cover state.

**Figure 5:**
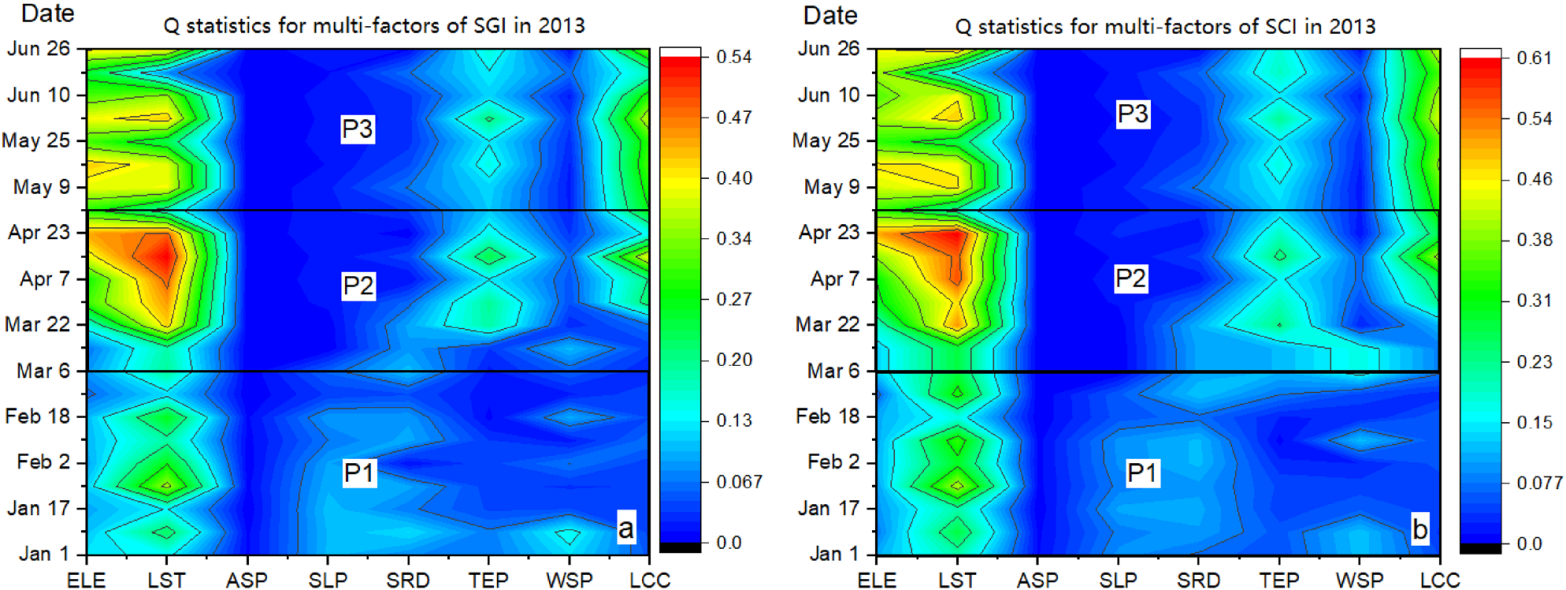
Variations in Q statistics for multi-factors of SGI (6.a) and SCI (6.b) in 2013. Map created in OriginPro, Version 2021. OriginLab Corporation, Northampton, MA, USA. (https://www.originlab.com/).

**Figure 6:**
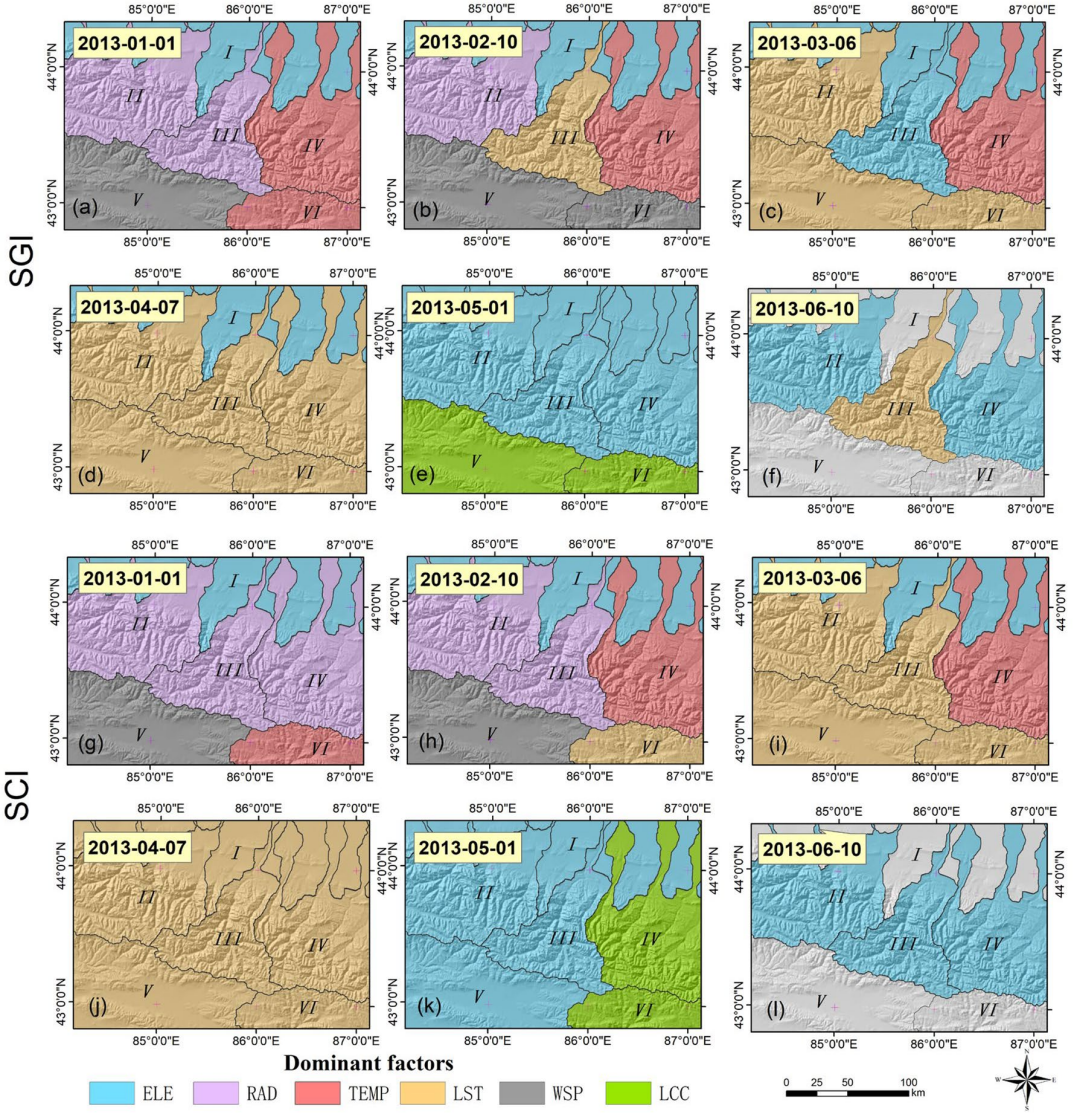
Spatial distribution of dominant regional factors for SGI and SCI in 2013. I–VI represent the subregions extracted after combining watershed terrain features based on the digital elevation model. Map created in ArcMap 10.6 of the Environmental System Resource Institute, Inc. (https://www.esri.com/software/arcgis/arcgis-for-desktop).

Figure 5 shows the detailed analysis of the effects and trend of variations of multiple factors on the SGI and SCI of 2013. In general, the impact and variation trend of each factor on the SGI and SCI are highly similar (Fig. 6a,b).During ablation period I, the effect of LST was significant with a trend of slackening increase, whereas ELE had a weak but stable effect. Note that the WSP effect was non-negligible at this stage. In addition, SRAD and SLP had a continuous and obvious effect on snow cover change during this period.

In ablation period II, the dominant impact factors were LST, ELE, TEMP, and LCC. The effect of these four factors increased over time, whereas that of other factors significantly decreased. In ablation period III, the effects of LST, ELE, and TEMP exhibited fluctuating changes, whereas the degree of the effects was reduced compared with that in ablation period II. However, the effect of LCC significantly increased.

#### Local influencing factors in the snow melting process

Figure 6 shows the spatial distribution of the dominant regional factors affecting the SGI and SCI in six sub-basins. The results indicate that the spatial distribution of dominant factors for the SGI and SCI are generally similar.

As Fig. 6a,b,g,h shows, in the early stage of ablation, SRAD was the dominant factor causing snow change in the northern and central alpine areas (*Q*_RAD_ = 0.23). LST and TEMP had weak and similar effects in these regions (*Q*_Temp_ = 0.04, *Q*_LST_ = 0.10). However, in the southern plain of the study area, WSP was the most significant leading influencing factor (*Q*_wsp_ = 0.149).

During the mid-melting period, the northern part of the study area was primarily controlled by ELE, LST, and TEMP. Over time, most of the study areas became controlled by LST (*Q*_LST_ = 0.40) and ELE (*Q*_ELE_ = 0.36) (Fig. 6c,d,i,j).

By the end of the ablation period, the effect of ELE gradually expanded from north to south. LCC (*Q*_LCC_ = 0.38) became the dominant factor in the south of the study area, whereas the east of the study area was mainly controlled by LST (*Q*_LCC_ = 0.26) (Fig. 6e,f,k,l).

This may be because in the pre-melting stage, even if at very low temperature, SRAD caused a change in the snow cover status. Because of the wind tunnel effect formed by the east–west mountain orientation, high wind speeds also caused a change in snow cover. During the mid-melting period, the increase in LST and TEMP accelerated melting. The snow cover on the northern slope was more significantly affected by ELE owing to the effect of topographic uplift. In the post-stage of ablation, the overall temperature rise caused the change in the snow cover rate to be more affected by LCC and ELE.

### Interactions of impact factors in snow melting

#### Overall interactive effect of multiple factors in snow melting

Table 4 shows the average values of interactions between pairs of factors listed for three ablation periods. These values are based on the interaction results of the snow grain size and contamination index from 2013 to 2017. During P1, there was no significant interaction between factors, with ELE and RAD having the most significant interactive effect. Cross interactions were minimal for ELE, TEMP, WSP, and LST. In P2, the interactions between TEMP and LCC significantly increased, as did the interactions between ELE, RAD, LST, and WSP. The interaction degree for each factor in P3 significantly increased at the end of ablation. The cross interactive effects between ELE, RAD, SLP, TEMP, and LCC were all distinctive. Notably, the interaction of SLP became more prominent at the end of ablation in this period. During the three periods, most factors exhibited nonlinear interaction, and the interaction types of ELE ∩ ASP, LST ∩ ASP, and ASP ∩ SLP were bilinear.

**Table 4:**
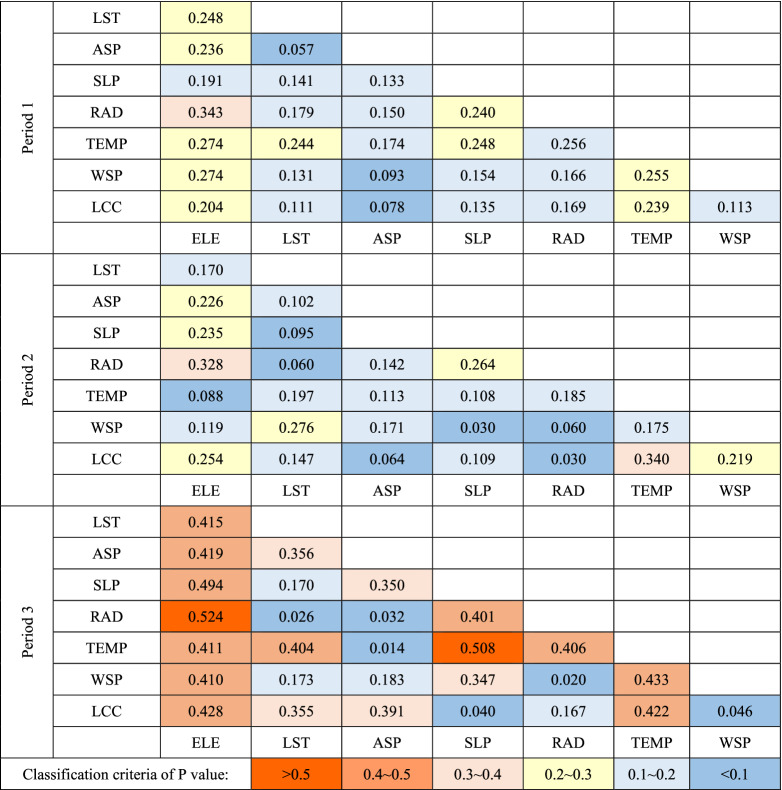
Q values of the interaction between of snow-melting factors in three periods.

The warm and cool colors are used to distinguish the *Q* and *P* values. The warmer the color is, the more important it is, and vice versa.

#### Regional differences in interactions of multiple factors

The *Q* value of each of the interactive pair of factors was found to be larger than the *Q* values of each of the two factors. Some of the *Q* values of the interactive pairs of factors were even larger than the sum of the *Q* values of the individual factors.

5 $$P_{factor1,2} = Q_{Interaction} - Q_{factor1} - Q_{factor2}$$

The parameter $P_{factor1,2}$ was proposed here to quantify the interaction between two factors. Its value is the difference between the *Q* values of the stacked factors and the sum of the *Q* value of each factor. The regional maximum *P* values in different periods were statistically analyzed to explore the temporal and spatial differentiation characteristics of the environmental factor interactions. Table 5 shows three pairs of interactive factors with the most significant interaction in different subregions for the three stages of melting with their corresponding *P* values.

**Table 5:**
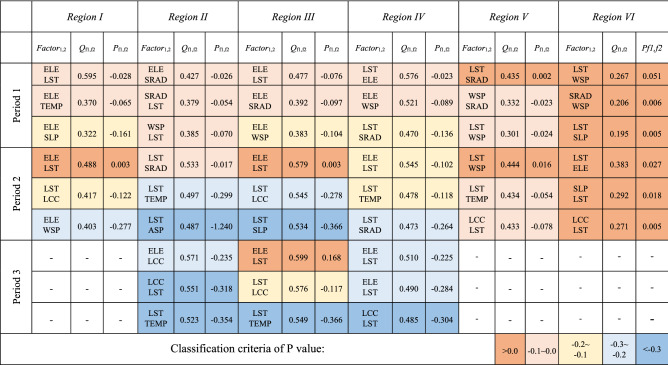
Top three *P* values of regional multi-factor interaction during the three periods.

The warm and cool colors are used to distinguish the *Q* and *P* values. The warmer the color is, the more important it is, and vice versa.

We found that ELE and LST always had the highest interactions with other factors, which was determined by comparing the interactive factor pairs with high *Q* values. Each subregion was found to have pairwise enhancing effects among the major dominant impact factors. The types of interaction enhancement and the degree of interaction vary in each subregion. In the early stage of snow melting, ELE interacted with LST, RAD, WSP, and SLP in subregions I, II, III, and IV. However, in subregions V and VI, the interactions between LSP, RAD, and SLP were significant, with a higher enhancement effect. In the mid-ablation period, the main interaction in each region was between ELE and LST, and the enhancement effect of multi-factor interaction was enhanced in all regions. The interaction enhancement effect within regions V and VI was significantly greater than that in other regions.

Even though the interaction of several components had weakened by the end of the melting period, ELE and LST still had significant interaction. Note that the interaction between LCC and other factors is more prominent at the end of ablation. Despite the proportion of forest being small, the snow cover at the end of ablation was concentrated in mountainous areas with a significant vertical difference. The surface vegetation type with remaining snow cover included grassland, forestland, barren and sparse vegetation, snow, and ice.

## Discussion

### Comparison with previous studies

**Figure 7:**
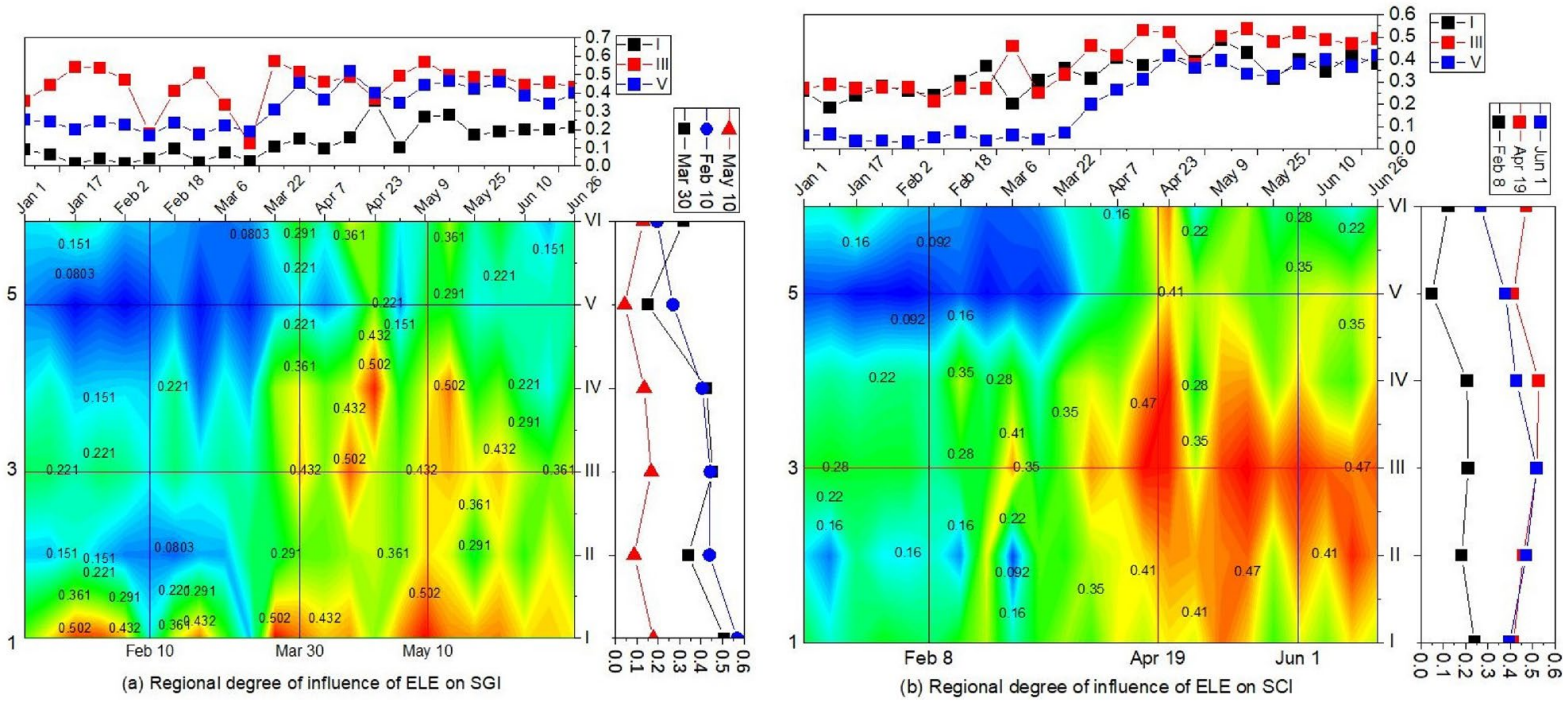
Spatiotemporal variations in the regional degree of influence of ELE on SGI and SCI. Map created in OriginPro, Version 2021. OriginLab Corporation, Northampton, MA, USA. (https://www.originlab.com/).

Many studies have addressed the effect of uplifted terrain on snow cover, determining that with increasing elevation, the negative feedback of air temperature increases, particularly at elevations over 2000 m a.s.l^10,47^. A threshold altitude of 3650 ± 150 m was found for the upper Heihe River Basin, below which air temperature is a negative factor in the SCA^18^. All these findings suggest the elevation’s prominent role and complex patterns in snowmelt. As shown in Fig. 7a,b, we analyzed variations in the effect of ELE in regions from north to south. It can be observed that (1) in subregion I located on the northern piedmont slope, ELE has a greater influence on snow melting in each period; (2) in the central mountainous area represented by subregion III, altitude has a lesser overall degree of influence, and the degree of influence in the early stage of ablation is significantly weaker than that in the final stage; (3) in subregion V, altitude has the lowest degree of influence, but it gradually increases with ablation. In general, altitude has the highest impact on the piedmont slopes in the north. It gradually decreases from north to south in the central mountainous areas and in the low-elevation areas in the south.

We further found that the solar radiation and wind speed were the dominant snow-melting factors in the northern and southern parts of the study area, respectively. This phenomenon is particularly noticeable during the pre-melting period. This is because the solar radiation, as the important component of the download radiation, promotes a climate by warming land temps. Whereas the heat that near the earth surface mainly comes from the download long-wave radiation, which is manifested as the rise of surface air temperature and land surface temperature. However, the amount of the download longwave radiation is not enough to cause a significant rise of the near-surface temperature or air temperature during the pre-melting period. Thus, the solar radiation is more prominent at this period. Moreover, the effect of westerlies can be seen by noting the wind channel effect formed within regions V and VI that are adjacent to a raised mountain range in regions II, III, and IV. This is because the enhanced downward sensible heat flux to snowpack is mainly due to the enhanced surface heat exchange coefficient induced by high surface wind speeds. Wind speed was confirmed to increase the latent heat flux of snow, causing sublimation at the snow surface^48^.

During the rapid ablation period, TEMP, LST, and ELE are the main factors that accelerate ablation in all regions, with a significant degree of influence. Factors such as topography would significantly redistribute the net surface radiation. The effect of elevation was primarily present in the northern area owing to the uplift along the north slope, with a significant increase in the degree of the effect. Whereas the enhanced ground energy absorption is driving by both the download longwave radiation (e.g. rising in air and surface temperature) and strong melting-induced snow cover reduction. The reduction of snowcover significantly decreased the surface albedo that can lead to the enhancement of short-wave radiation absorption on snowcover surface, which can accelerate snowmelting. In previous studies, the air temperature was found to be the most influential factor during this period; however, our findings show that the influence of surface temperature is more significant. Compared with the near-surface temperature, the snow surface temperature is more likely to rise owing to the absorption of download radiation^49^.

In comparison, the influence of the underlying surface type on snow ablation was more prominent in the late stage of snow melting. We determined that the snow cover at lower altitudes had completely melted during this period, and the remaining snow cover was mostly distributed among the mountainous areas. Thus, the enhanced interactions between LCC and other hydrothermal factors are more complex in regions with distinct land cover types. Compared with the influencing factors in snow surface evaporation, different energy storage types in different underlying surfaces will alter the snow ablation patterns in various regions. Snow ablation is influenced by vegetation through complex snow–canopy interactions such as canopy interception, solar radiation blocking, longwave canopy radiation, wind attenuation, and below-canopy turbulence^50–52^. A significant elevation drop in mountainous areas caused continuous snowmelt runoff, which further accelerated the snow-melting process.

The combined findings verified the effect of net radiation on the snow melting process. The melting results from the enhanced downward sensible heat flux to snowpack and enhanced ground solar radiation absorption, with generally larger contributions from the former at the pre-melting period and from the latter after that. Undulating terrain has a significant effect on energy redistribution, resulting in local differences in radiation, temperature, wind speed, surface type, and other conditions. The combined action of these local factors directly or indirectly affects the heating of the shallow surface and the heat storage conditions under the snow cover, affects changes in the snow cover state in the region, and demonstrates the heterogeneity of the factors that influence snow cover in different temporal and spatial regions.

### Limitations and further work

This paper aims to quantify the degree of influence of different factors in the distribution of snow status using the geographical detector method. Additional local factors, such as rainfall, river distance, and soil water content of the underlying surface should be considered. Because of poor data quality in the study area, precipitation data were not included. The inconsistent spatial resolutions of various impact factor data will also have an impact on the detection results. For example, although both air temperature and LST are considered in this study, the spatial resolution of air temperature data is still lower than that of LST, which may be the reason for the low spatial correlation between air temperature and snow cover, suggesting that the difference in coupling between air temperature and LST and snow melting merits further investigation.

In addition, analyses performed on different scales may lead some factors to be neglected. For example, the impact of local topography is particularly prominent on a small scale, which is difficult to represent in a large-scale analysis. The varying characteristics of the influencing factors at different scales and the disturbance effects during the ablation process remain a topic for further study.

In the current work, only the snow grain size and contamination index based on optical data were used as dependent variables, and parameters more closely related to snow ablation, such as snow depth, snow humidity should also be selected. To provide better decision-making support for regional snow ablation and runoff simulations, the influence of local factors on the distribution of annual snow ablation was studied by comprehensively considering problems at different scales.

## Conclusions

This study combined the SGI and SCI as dependent variables and multiple environmental influencing factors as independent variables. The geodetector method was used to quantitatively detect the response of snow melting status parameters to various environmental factors during the melting seasons from 2013 to 2017. Spatiotemporal partitioning was used to explore the regional response to influencing factors and their interactions during different melting periods. The conclusions are as follows:

1. By dividing changes in the snow cover status into periods, we observe similar responses of the snow grain size index and contamination index to multiple influencing factors. On the overall scale, the variation characteristics of the factors that influence snow melting have commonalities across different years, indicating a regular pattern in the influencing factors of snow ablation in the study area in recent years.
2. In the period before ablation, the snow cover state also changed. LST became the dominant influencing factor during this period on a overall scale. In the rapid melting period, LST and ELE were the main factors affecting snow cover decline. This indicates that temperature still affects snow cover on a large scale, while terrain has a significant effect on the redistribution of spatial heat distribution.
3. As the scale decreases, local factors such as radiation, slope, and surface type become more prominent. Before the ablation period, SRAD and LST were the dominant factors in the northern part of the study area, while wind speed was the dominant factor in the southern part. During the rapid ablation period, ELE, LST, and TEMP acted together from north to south. At the end of the ablation period, complete snow melt occurred at low altitudes, and the snow status was more closely reflected in the high correlation with LCC. The influence of a certain factor on annual snow melt also differed significantly in different study areas, with the difference being related to the distribution characteristics of that factor in different regions.
4. Regardless of the interaction between overall and local factors, the results show that the interaction between two variables has a greater impact on snow cover distribution than that of a single variable. The interaction enhancement effect can be double factor enhancement or nonlinear enhancement. The factor pairs with the highest interaction enhancement results are also those whose influencing factors are predominant in the local space.

## Data Availability

The datasets analyzed during the current study are available in the table below.

**Table:** 

Name	Download links
MOD09GA	https://ladsweb.modaps.eosdis.nasa.gov/
MOD10A1	http://www.crensed.ac.cn/portal/
ASTER G-DEM	http://gdem.ersdac.jspacesystems.or.jp/
MCD12Q1	https://ladsweb.modaps.eosdis.nasa.gov/
AWLSTD	https://data.tpdc.ac.cn/zh-hans/
CMFD	https://data.tpdc.ac.cn/en/data
